# Deep analysis of neuroblastoma core regulatory circuitries using online databases and integrated bioinformatics shows their pan-cancer roles as prognostic predictors

**DOI:** 10.1007/s12672-021-00452-3

**Published:** 2021-11-29

**Authors:** Leila Jahangiri, Perla Pucci, Tala Ishola, Joao Pereira, Megan L. Cavanagh, Suzanne D. Turner

**Affiliations:** 1 Department of Life Sciences, Birmingham City University, Birmingham, UK; 2 School of Science & Technology, Nottingham Trent University, Clifton Lane, Nottingham, NG11 8NS UK; 3 Division of Cellular and Molecular Pathology, Addenbrooke’s Hospital, University of Cambridge, Cambridge, UK; 4 Department of Neurology, Massachusetts General Hospital, Boston, MA USA; 5 CEITEC, Masaryk University, Brno, Czech Republic

**Keywords:** Core regulatory circuitry, Neuroblastoma, Solid cancers, Tumour microenvironment, Gene networks, Differentiation

## Abstract

**Aim:**

Neuroblastoma is a heterogeneous childhood cancer derived from the neural crest. The dual cell identities of neuroblastoma include Mesenchymal (MES) and Adrenergic (ADRN). These identities are conferred by a small set of tightly-regulated transcription factors (TFs) binding super enhancers, collectively forming core regulatory circuitries (CRCs). The purpose of this study was to gain a deep understanding of the role of MES and ADRN TFs in neuroblastoma and other cancers as potential indicators of disease prognosis, progression, and relapse.

**Methods:**

To that end, we first investigated the expression and mutational profile of MES and ADRN TFs in neuroblastoma. Moreover, we established their correlation with neuroblastoma risk groups and overall survival while establishing their extended networks with long non-coding RNAs (lncRNAs). Furthermore, we analysed the pan-cancer expression and mutational profile of these TFs and their correlation with patient survival and finally their network connectivity, using a panel of bioinformatic tools including GEPIA2, human pathology atlas, TIMER2, Omicsnet, and Cytoscape.

**Results:**

We show the association of multiple MES and ADRN TFs with neuroblastoma risk groups and overall survival and find significantly higher expression of various MES and ADRN TFs compared to normal tissues and their association with overall survival and disease-free survival in multiple cancers. Moreover, we report the strong correlation of the expression of these TFs with the infiltration of stromal and immune cells in the tumour microenvironment and with stemness and metastasis-related genes. Furthermore, we reveal extended pan-cancer networks comprising these TFs that influence the tumour microenvironment and metastasis and may be useful indicators of cancer prognosis and patient survival.

**Conclusion:**

Our meta-analysis shows the significance of MES and ADRN TFs as indicators of patient prognosis and the putative utility of these TFs as potential novel biomarkers.

**Supplementary Information:**

The online version contains supplementary material available at 10.1007/s12672-021-00452-3.

## Introduction

Neuroblastoma (NB) is a paediatric malignancy that accounts for circa 15% of cancer-related paediatric deaths [1]. Based on clinical presentation, behaviour, and therapy response, this disease is classified into five stages (1–4 and 4S), with advanced stages (3 and 4) displaying metastatic behaviour [1, 2]. Risk groups are classified according to a variety of criteria, which include MYCN amplification status, age, disease stage, histologic characteristics, grade of tumour, ploidy, and chromosome 11q structural alterations. For instance, the presence of MYCN amplification, poor differentiation status, diploidy, and chromosome 11q aberrations are associated with unfavourable outcomes. Indeed, NB cases with MYCN amplification, representing ~ 50% of patients in high-risk groups, have a 5-year survival of 40–50% [1–3]. NB is formed of cells of the neural crest that are halted in their developmental stages and fail to differentiate [4]. Previous studies have identified the dominance of epigenetic regulation in this form of cancer, establishing tumour identities inclusive of two cell types. Accordingly, tumours are classified by the prevalence of neural crest migratory (mesenchymal, MES) and committed adrenergic (ADRN) cellular sub-types [5–7]. Notably, cell identity can be conferred by tightly regulated transcription factors (TFs) which engage super enhancers genome-wide, collectively forming core regulatory circuitries (CRCs) [8–11]. These TFs self-regulate and bind to regulatory regions of other CRC TFs, driving lineage-specific gene expression, hence cell identity [8–11]. NB ADRN subtype-specific genes include *PHOX2B, PHOX2A,* and *DBH,* while the MES subtype expresses high levels of *SNAI2, FN1,* and *VIM* [5–7]*.* The extended MES and ADRN CRCs comprise 485 and 369 genes, respectively, a list further refined by van Groningen and colleagues to just 20 MES and 18 ADRN CRC TFs that constitute the core TFs [5, 6]. The role of these TFs in NB development and maintenance of their normal cell counterparts has been studied to a certain extent. For instance, the ADRN CRC TF, ASCL1, a bHLH TF is implicated in cell growth and differentiation arrest, while GATA3 is a biomarker linked to suppression of differentiation. Furthermore, ISL1 suppresses genes that are involved in the process of NB differentiation [12–14]. Conversely, PHOX2A and PHOX2B are indicated in neural progenitor differentiation [15], while HAND1, SOX11, and TFAP2B are crucial for differentiation towards catecholaminergic, sympatho-adrenergic, and adrenergic fates, respectively [16–18]. Hence, these TFs not only play a major role in the developmental processes of neural differentiation toward specific sympathoadrenal fates, processes that diverge from their normal developmental programme in NB, but also govern the establishment of MES and ADRN identities in NB.

From the viewpoint of patient prognosis, the association of subsets of these TFs with NB risk groups, patient survival, prognostic and diagnostic indications in NB has been investigated [13, 19–26], although not extensively for all of the TFs. Furthermore, many of these CRC TFs may be implicated in gene regulatory networks, molecular pathways, and signalling cascades common to various cancers. Therefore, they may have prognostic value in NB and form extended networks with long non-coding RNAs (lncRNAs). LncRNAs are often upregulated in cancer and are increasingly characterised as potential prognostic and diagnostic biomarkers and therapeutic targets for several cancers, including NB [20]. Moreover, we extended these studies to their expression and association with patient survival across various cancers.

In NB, T-cell infiltration to the tumour microenvironment (TME), correlates with enhanced patient overall survival (OS). Specifically, the expression and activation of MYCN, ASCL1, and SOX11 are inversely correlated with T-cell infiltration in NB and correlate with patient OS [27], supporting the significance of these TFs in the NB TME. Notably, similar associations between MES and ADRN TFs with the infiltration of cancer-associated fibroblasts (CAFs) of the TME implicate these TFs in pro-tumourigenic remodelling of the TME in cancers [28–31]. Furthermore, tightly associated with these events are epithelial to mesenchymal transition (EMT) and cancer stemness markers, including CD44, CDH1, CDH2, FN1, FOXC2, NANOG, SOX2, TWIST1, and VIM that are triggered by hypoxia in the TME [32, 33]. Given this background, we were intrigued to conduct a comprehensive pan-cancer assessment of the association of these TFs with the infiltration of tumour-associated immune and stromal cells in the TME. We also focused on the link between these TFs with markers of EMT and stemness and the resulting extended mRNA-microRNA (miRNA) regulatory networks they form. For this, we have leveraged multiple tools including cBioPortal, GEPIA2, the human pathology atlas, TIMER2, Omicsnet, and Cystoscape. We report the misregulation of a subset of both MES and ADRN TFs in NB high-risk groups, their correlation with patient survival, and intricate gene regulatory networks. In a further pan-cancer approach, we report the high expression of MES and ADRN TFs in multiple cancer types compared to normal tissue, the association of this expression pattern with prognosis, and altered OS and disease-free survival (DFS). Furthermore, we identify an association between cancer-associated fibroblasts (CAFs), T regulatory cells (Tregs), B cells, gamma-delta T cells, endothelial cells (ECs) of the TME, and markers of EMT and stemness with the expression of MES and ADRN TFs and their connectivity with other cancers and TF networks. Finally, we report the role of MES and ADRN TFs as indicators of patient survival in NB and other solid cancers.

## Materials and methods

### Gene Ontology of MES and ADRN genes and their correlation with NB risk groups

A list of 485 MES and 369 ADRN genes was subjected to Gene Ontology enrichment analysis using PANTHER (Additional file 1: Materials S1) [5–7, 34]. Despite the paucity of somatic mutations in NB and the importance of epigenetic regulation in this cancer [35], we subjected 854 MES and ADRN extended CRC network genes to the cBioportal genetic alteration tool, having selected 1472 NB patient samples including AMC Amsterdam, Nature 2012 (87 samples), Broad, Nature Genetics 2013 (240 samples), Broad, Nature 2015 (56 samples) and Paediatric NB, TARGET, 2018 (1089 samples) (Additional file 1: Materials S1) [36, 37].

Furthermore, we narrowed our analysis to the 20 MES and 18 ADRNs TFs, as reported by van Groningen and colleagues, since these TFs comprise the core defining factors of MES and ADRN identities and include ELK4, CREG1, DCAF6, ID1, SMAD3, SIX4, SIX1, MAML2, NOTCH2, CBFB, IFI16, ZNF217, EGR3, ZFP36L1, WWTR1, PRRX1, SOX9, MEOX1, MEOX2, AEBP1, for MES and ZNF536, PHOX2A, HAND1, ASCL1, KLF13, SOX11, GATA2, GATA3, KLF7, EYA1, TFAP2B, ISL1, HEY1, SIX3, DACH1, PHOX2B, PBX3, SATB1 for ADRN [5–7]. For simplicity, hereafter, we have referred to these TFs as “the 38 TFs”. To assess the correlation of MES and ADRN TF expression with low/intermediate and high-risk groups of NB patients, we used TARGET data deposited to cBioPortal. Low-risk NB patients were infants younger than 18 months lacking MYCN amplification with either localised or metastatic disease. Half of the high-risk cases displayed MYCN amplification, while the remaining half may have displayed a combination of other high-risk associated criteria [1]. In addition, event-free survival (EFS), the time after the primary treatment in which the patient did not experience any events that the treatment aimed to delay or prevent, with a cut-off of 75–85%, 50–75%, and < 50% were defined as low, intermediate, and high-risk NB cases, respectively [3].

The expression correlation analyses were performed on cBioPortal RNA sequencing data of 143 NB patient samples (TARGET, 2018) for the 38 MES and ADRN genes. The top 20 positively correlated genes were shortlisted for each of the MES and ADRN genes in order to select the strongest co-expression correlations. Amongst them, 9 lncRNAs positively correlated with 10 ADRN and MES genes, most of which with strong correlations (Spearman > 0.7).

The correlation between TFs and lncRNAs expression with patient OS was obtained from cBioPortal and the following method was used: the samples were grouped based on the upregulation (high) or downregulation (low) of each queried gene, using z-score equals 1 or a consistent criterion of at least 10% of samples displaying upregulation and relevant survival plots were generated. In this statistical test, the variable of interest was the time elapsed before event occurrence. Accordingly, increased and decreased OS accounted for an elongated or shortened duration for which the patient was alive.

### Gene expression analysis

Expression profiles of the 38 TFs were investigated in more than 30 cancer types using TCGA (tumour) and matching normal tissue data using the GEPIA2 expression DIY module [38]. This programme collated expression data from tumour and normal tissue as the log2 fold change (log2FC) with a cut-off of 1 and *p* < 0.01 and used one-way ANOVA to assign statistical significance. Cancer name abbreviations have been provided in the list of abbreviations. We also linked the expression of the 38 TFs in over 30 cancer types with their mutational profile in these cancers using cBioPortal (Additional file 1: Materials S2).

### Independent prognostic summary

The 38 TFs were further studied using TIMER2 across over 30 cancer types to determine the clinical relevance of their expression [39, 40]. This tool used a Cox proportional hazard (PH) evaluation to assess the clinical significance of the expression of a gene in a cancer type and provided Kaplan Meier OS analyses.

Furthermore, OS and EFS maps and Kaplan Meier curves were generated using the GEPIA2 tool, which also used the COX PH model. OS and EFS represented the time for which a patient was alive and the duration for which the patient did not display signs/ symptoms of cancer, respectively. The median expression was utilised as a cut-off between low and high expression, with the number of patient tissue samples per cancer type reported in Additional file 1: Materials S3 (although this tool did not specify whether these samples were primary or secondary tumours).

### The prognostic value of expression of the 38 TFs

The prognostic significance of the 38 TFs was investigated across multiple cancer types using the human pathology atlas (https://www.proteinatlas.org/). The classification of favourable and unfavourable prognoses in this database is based on Kaplan Meier survival analyses.

### The correlation between immune cell infiltration and gene expression across cancer types

The 38 TFs were further studied using TIMER2 for immune cell infiltration correlations for over 30 cancer types [39, 40]. TIMER2 utilised deconvolution statistical methods to determine the distribution of tumour infiltrating cells in the context of TCGA gene expression profiles. Partial Spearman's correlation allowed for the assessment of immune cell infiltration with adjustment for tumour purity. The number of patient tissue samples utilised for each cancer is reported in Additional file 1: Materials S4 (this tool did not specify whether these samples were primary or secondary tumours).

### The association of the 38 TFs with EMT and cancer cell genes across cancer types

The TIMER2 gene correlation module was used to establish the association of MES and ADRN TFs with a list of genes involved in EMT and cancer stemness markers including CD44, CDH1, CDH2, FN1, FOXC2, NANOG, SOX2, TWIST1, and VIM [32].

### Omicsnet, Cytoscape, and dbCorC database for network analysis for the 38 TFs

Omicsnet was used to investigate different types of network interactions [41]. We used the 38 TF gene IDs and built networks using default settings (transcription factor gene interactome; TGI) and 2D visualisation. TGI utilised the information in TRRUST, JASPAR, and ENCODE databases and the network was subjected to enrichment analysis by selecting the KEGG gene option. Further, we utilised Cytoscape 3.8.0 [42] and NDEx v2.4.5 [43] to investigate and visualise the mRNA versus miRNA networks of GATA3 and SOX9 in hepatocellular cancer (HCC) and diffuse large B cell lymphoma (DLBCL), respectively.

Finally, the dbCoRC database was utilised to integrate the mRNA expression of core TFs with their reconstructed circuitry. This database archived information about CRC components, including CRC TFs, binding sites for TFs, and super enhancer genomic coordinates, allowing for the interrogation of specific TFs in CRCs of defined cell types and the subsequent visualisation of the CRCs constructed [44].

## Results

### MES and ADRN TFs are associated with NB risk groups, patient survival, and lncRNA expression

Gene Ontology analysis was conducted on the 485 MES and 369 ADRN genes previously reported to define the respective NB cell subtypes [5–7]. Analysis of ADRN genes yielded the term ‘enrichment for adrenaline and dopamine-related pathways’, while for MES, terms such as ‘angiogenesis’, ‘cadherin’, ‘PDGF’, ‘integrin’, ‘JAK/STAT signalling pathways’, and ‘inflammatory pathways’ were obtained (Additional file 1: Materials S1) [45].

cBioPortal analysis was conducted of 1472 individual NB primary patient tissues previously included in large studies (i.e., Neuroblastoma (AMC Amsterdam), Neuroblastoma (Broad 2013 and 2015), Paediatric Neuroblastoma (TARGET)) using the 485 MES and 369 ADRN associated genes revealing genetic alterations of putative drivers, e.g., *EPHA3 A629* splice mutation, *FGFR1 N546K* missense mutation and *LATS2 P479_A480insPP* (Additional file 1: Materials S1). *EPHA3 A629* splice mutation, *per se*, was identified in sample NBL44 data deposited to cBioPortal and is likely oncogenic, while *FGFR1 N546K* missense mutation was identified in TARGET-30-PARCRR sample revealing an allele frequency of 0.47, represented in less than 1% of the TARGET cohort. Finally, *LATS2 P479_A480insPP* identified in NBL35 sample is an in-frame mutation that is predicted to be oncogenic. Apart from these predicted drivers, we have also linked each genetic alteration with unknown significance reported in Additional file 1: Materials S1, with specific identifiers, which will allow obtaining more information about the alteration and the corresponding patient data. For instance, a missense mutation of unknown significance, *DLC1 S978C*, is linked to TARGET-30-PAMVLG sample. The inspection of this alteration using cBioportal reveals that the allelic frequency is 0.55 and specific clinical information about the corresponding patient from which this sample was obtained. These data are significant, since, apart from *MYCN* and *ALK* genetic alterations, others, especially those with a putative oncogenic driver description, have not previously been described in NB. These discoveries could provide a blueprint for future diagnostic and therapeutic endeavours.

Given these results, we sought to determine correlations between these TFs, risk groups, and patient survival. Multiple criteria influence high-risk stratification in NB, and for clarity, we have referred to the International Neuroblastoma Risk Group (INRG) classification, where EFS cut-offs are 75–85% (low-risk), 50–75% (intermediate-risk), and < 50% (high-risk) [3].

**Fig. 1:**
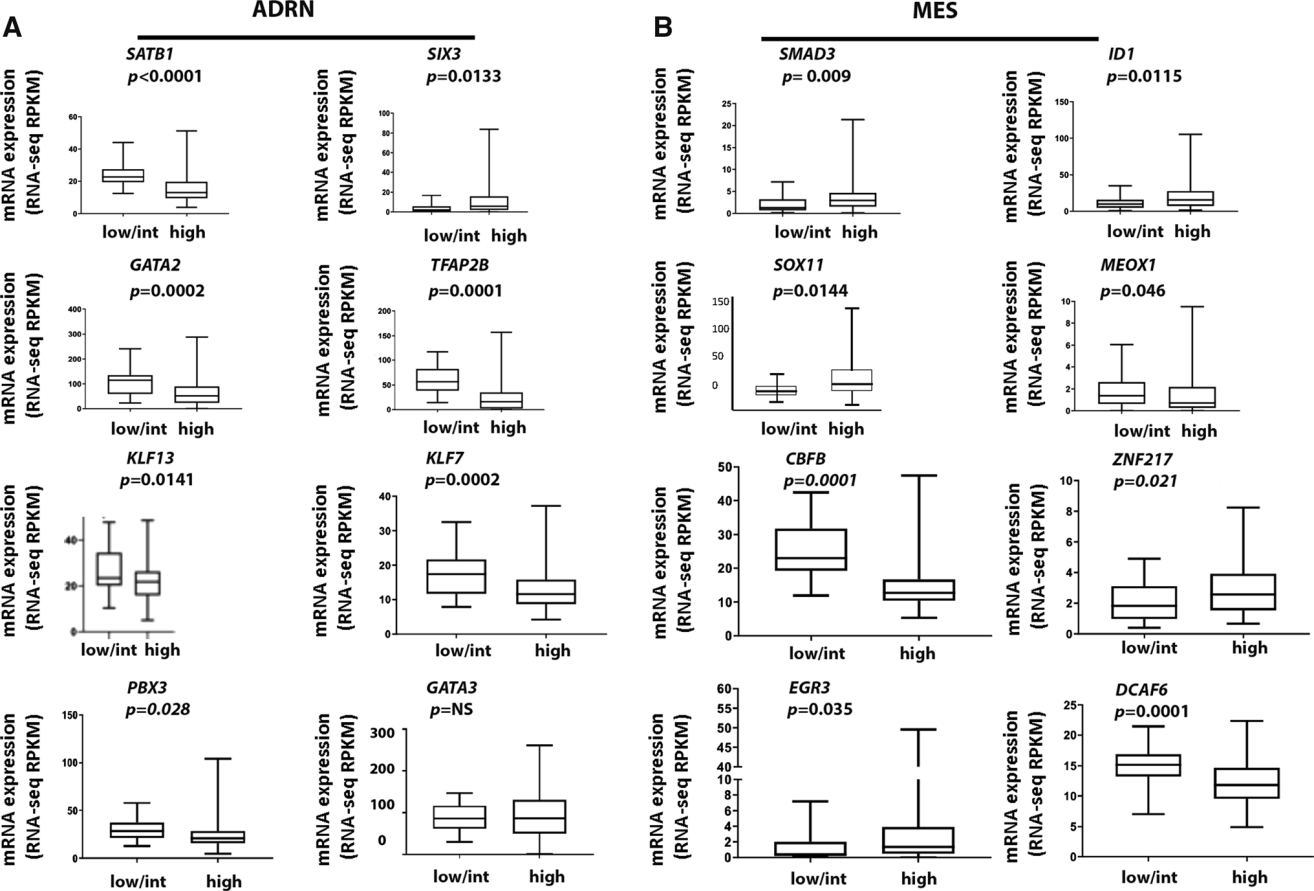
ADRN and MES TF expression correlates with NB risk group and OS. **A** 8 ADRN TFs significantly correlate with NB risk (student t-test). *SATB1*, *GATA2*, *TFAP2B, KLF13*, *KLF7* and *PBX3* are downregulated in high-risk NB cases, while *SIX3* and *GATA3* are upregulated in this group (although the latter is not significant). **B** 8 MES TFs significantly correlate with NB risk (student t-test). *MEOX1, CBFB* and *DCAF6* are downregulated in high-risk NB cases, while *SMAD3, ID1*, *SOX11, ZNF217* and *EGR3* are upregulated in the high NB risk group. Data shown are representative of 143 samples from NB tissue processed for RNA sequencing (TARGET, 2018). Low, intermediate, and high-risk cases have an EFS of 75–85%, 50–75% or < 50% respectively. NS: not significant

Upon correlating the refined list of 38 TFs with risk groups in NB patient samples, we report that 8 ADRN and 8 MES TFs were significantly up- or downregulated in high-risk NB, as defined by the previously mentioned parameters (Fig. 1A, B) [3]. These TFs include, for the ADRN group, *SATB1*, *GATA2*, *TFAP2B, KLF13*, *KLF7,* and *PBX3* that were downregulated in the NB high-risk group (*p*<0.0001, 0.0002, 0.0001, 0.0141, 0.0002, and 0.028, respectively). Conversely, *SIX3* and *GATA3* were upregulated in this group (*p* = 0.0133, not significant, respectively) (Fig. 1A).

Among the MES genes, *MEOX1, CBFB* and *DCAF6* were downregulated, (*p* = 0.046, 0.0001 and 0.0001, respectively), while *SMAD3, ID1*, *SOX11, ZNF217* and *EGR3* were upregulated in NB high-risk groups (*p* = 0.009, 0.0115, 0.014, 0.021 and 0.035 respectively) (Fig. 1B). Therefore, these TFs (isolated or in clusters) might represent biomarkers for assigning patients to risk groups for treatment stratification. Data shown in Fig. 1 are representative of 143 NB tissue samples analysed by RNA sequencing (TARGET, 2018).

Given these significant findings, we sought to follow up these results by assessing the correlation of these TFs with NB prognosis and patient survival. Of the CRC TF genes, 6 were associated with NB patient OS, based on reads per kilobase of transcripts per million reads (RPKM) values of RNA sequencing (Additional file 2: Fig. S1A) or Agilent microarray data (Additional file 2: Fig. S1B) deposited to cBioportal. For instance, *KLF7* and *TFAP2B* were upregulated in patients with increased survival. In contrast, *HAND1, EGR3, PBX3,* and *ASCL1* were upregulated in patients with reduced survival (although both *TFAP2B* and *HAND1* show trends) (Additional file 2: Fig. S1A, B). Notably, *KLF7* was associated with better survival outcomes based on both RNA sequencing and microarray analyses (Additional file 2: Fig. S1A, B). These data suggest a significant association of expression of these TFs with NB high-risk groups and their association with patient survival outcomes. However, these data will need to be validated in preclinical models and in a prospective clinical trial scenario, whereby a predictive outcome algorithm can be developed. These findings lay the groundwork for potential new biomarker discovery in both MES and ADRN subtypes of NB. A further factor that affects poor prognosis in NB is MYCN amplification status. Since a component of MYCN regulation and NB stratification depends on interactions with lncRNAs, we investigated potential links between lncRNAs with MES and ADRN TFs and NB features [46]

**Fig. 2:**
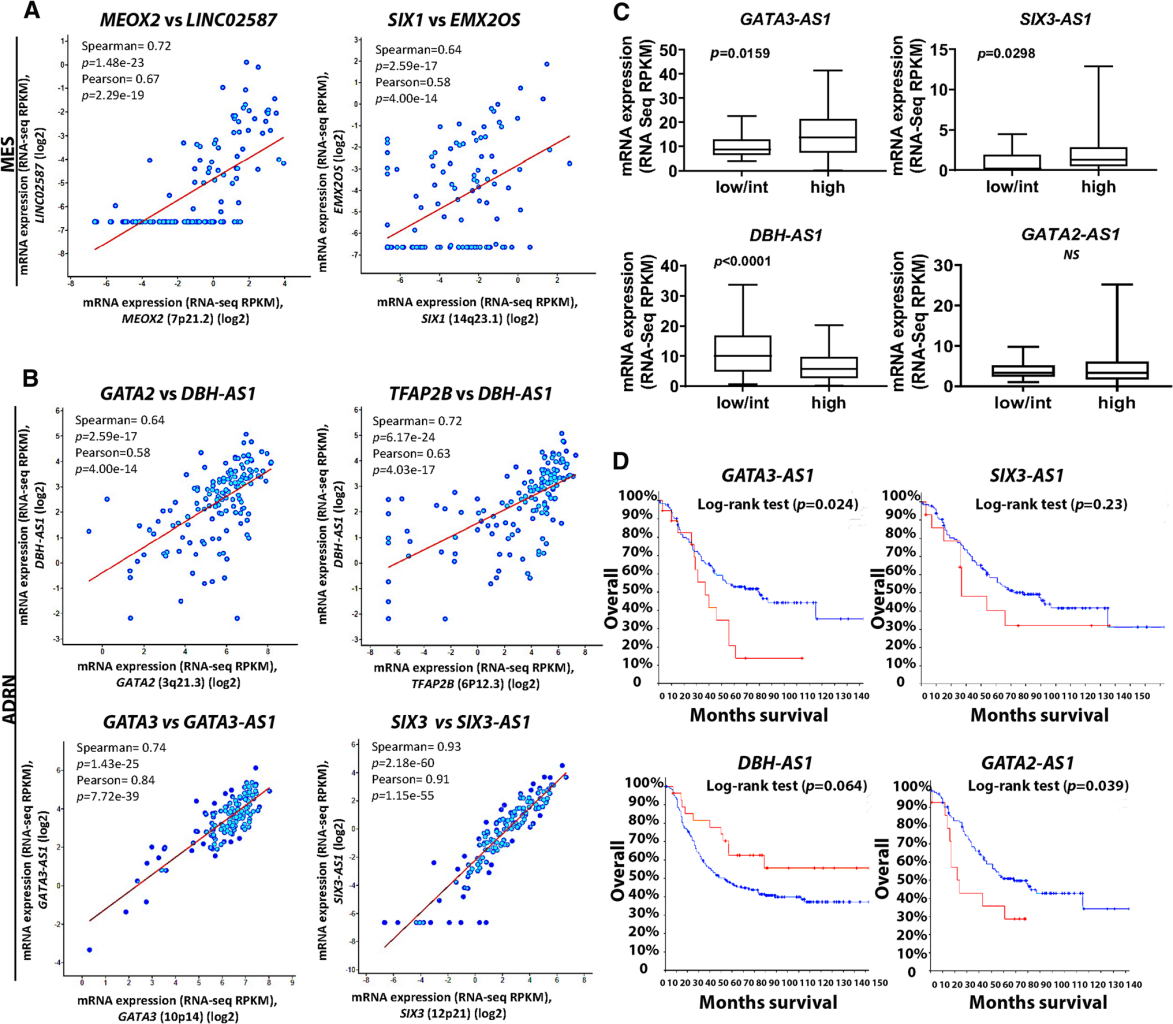
LncRNAs are co-expressed with MES and ADRN TFs and are associated with risk groups and survival of NB patients. **A**, **B** 6 TFs positively correlate with 6 lncRNAs, obtained from RNA sequencing data from 143 NB patient tissue samples (TARGET, 2018) (2 lncRNAs for MES (**A**) and 4 for ADRN (**B**) TFs). **C**, **D** LncRNAs are associated with NB risk group; for instance, *GATA3-AS1* and *SIX3-AS1* are overexpressed in high-risk NB (**C**) and survival in the same cohorts of patients. *GATA3-AS1* and *SIX3-AS1* are also upregulated in patients with lower OS, despite the latter not being significant (**D**) (red and blue lines represent increased and reduced expression, respectively). However, *GATA2-AS1* expression is not associated with NB groups, while it is upregulated in patients with reduced survival. *GATA2-AS1*, *GATA3-AS1* and *SIX3-AS1* survival data were obtained from RNA sequencing of 143 NB patient tissue, and *DBH-AS1* survival data were reported from Agilent microarray of 249 NB samples (TARGET, 2018). Statistics was calculated on mean ± SEM with student`s t test in (**C**). NS: not significant, SEM: standard error of mean

The correlation analyses of Spearman and Pearson found lncRNAs that positively correlate with MES and ADRN CRC genes (Fig. 2A, B and Additional file 2: Fig. S1C). For instance, *MEOX2, SIX1, GATA2, TFAP2B, GATA3, SIX3, PHOX2A, GATA2, SATB1,* and *TFAP2B* expression positively correlate with *LINC02587, EMX2OS, DBH-AS1, DBH-AS1, GATA3-AS1, SIX3-AS1, MORC2-AS1, GATA2-AS1, KLF9-AS1,* and *LIFR-AS1*, respectively. Notably*, DBH-AS1* expression strongly correlates with GATA2 (Spearman = 0.64, *p* = 2.59E-17) and TFAPB2 (Spearman = 0.72, *p* = 6.17E−24) (Fig. 2B).

Two other lncRNAs of particular interest were also identified, *SIX3-AS1* and *GATA3-AS1*, since their expression positively correlated with *SIX3* and *GATA3*, respectively (Fig. 2B). Both were upregulated in the NB high-risk group (*SIX3-AS1 p* = 0.0298 and *GATA3-AS1 p* = 0.015) (Fig. 2C). *GATA3-AS1* was associated with reduced survival (*p* = 0.024), even though for *SIX3-AS1* the log-rank test was not significant and did not reveal an association with patient OS (Fig. 2D).

*DBH-AS1* expression was also decreased in the NB high-risk group (*p* < 0.0001) (Fig. 2C) and was upregulated in patients with increased survival, although this result was not statistically significant (as defined in the Methods section, log-rank Test (*p* = 0.0642) (Fig. 2D).

*GATA2-AS1* also positively correlated with the ADRN TF GATA2 (Spearman = 0.71, *p* = 4.10E−23) (Additional file 2: Fig. S1C). The expression of *GATA2-AS1* in risk groups was not different (*p* = not significant) (Fig. 2C), while it was significantly associated with reduced OS (log-rank test *p* = 0.039) (Fig. 2D). *GATA2-AS1*, *GATA3-AS1,* and *SIX3-AS1* survival data are reported from RNA sequencing of 143 NB patient tissues (TARGET, 2018), while *DBH-AS1* survival data are reported from 249 Agilent microarray analyses of NB samples (TARGET, 2018).

Other lncRNA, such as *LIFR-AS1* and *KIF9-AS1*, were downregulated in high-risk NB (*p *< 0.0001 and *p *< 0.0001, respectively) (Additional file 2: Fig. S1D). All the expression data presented for both TFs and lncRNAs and their NB risk correlation are displayed as RNA sequencing RPKM values. These findings suggested that MES and ADRN TFs are co-expressed with lncRNAs and display similar association patterns with NB risk and patient survival. Notably, lncRNAs can interact with TFs, mostly by stabilising them and promoting their downstream activity [47].

Accordingly, *DBH-AS1* has been linked to viral-mediated hepatocellular carcinoma [48], while *GATA3-AS1* is associated with poor prognosis in breast cancer via the *GATA3-AS1*/*miR-495-3p*/CENPU axis [49]. Furthermore, *LIFR-AS1* regulates invasion and metastasis in thyroid cancers [50], while the downregulation of *EMX2OS* is associated with poor patient prognosis in clear cell carcinoma of the kidney [51]. In addition, the association of these lncRNAs with the 38 TFs in NB and other cancers led us to profile the role of these TFs and their extended gene networks in cancers.

### Gene expression analysis across cancer types using GEPIA2 shows that MES and ADRN TFs are widely expressed in cancers

**Fig. 3:**
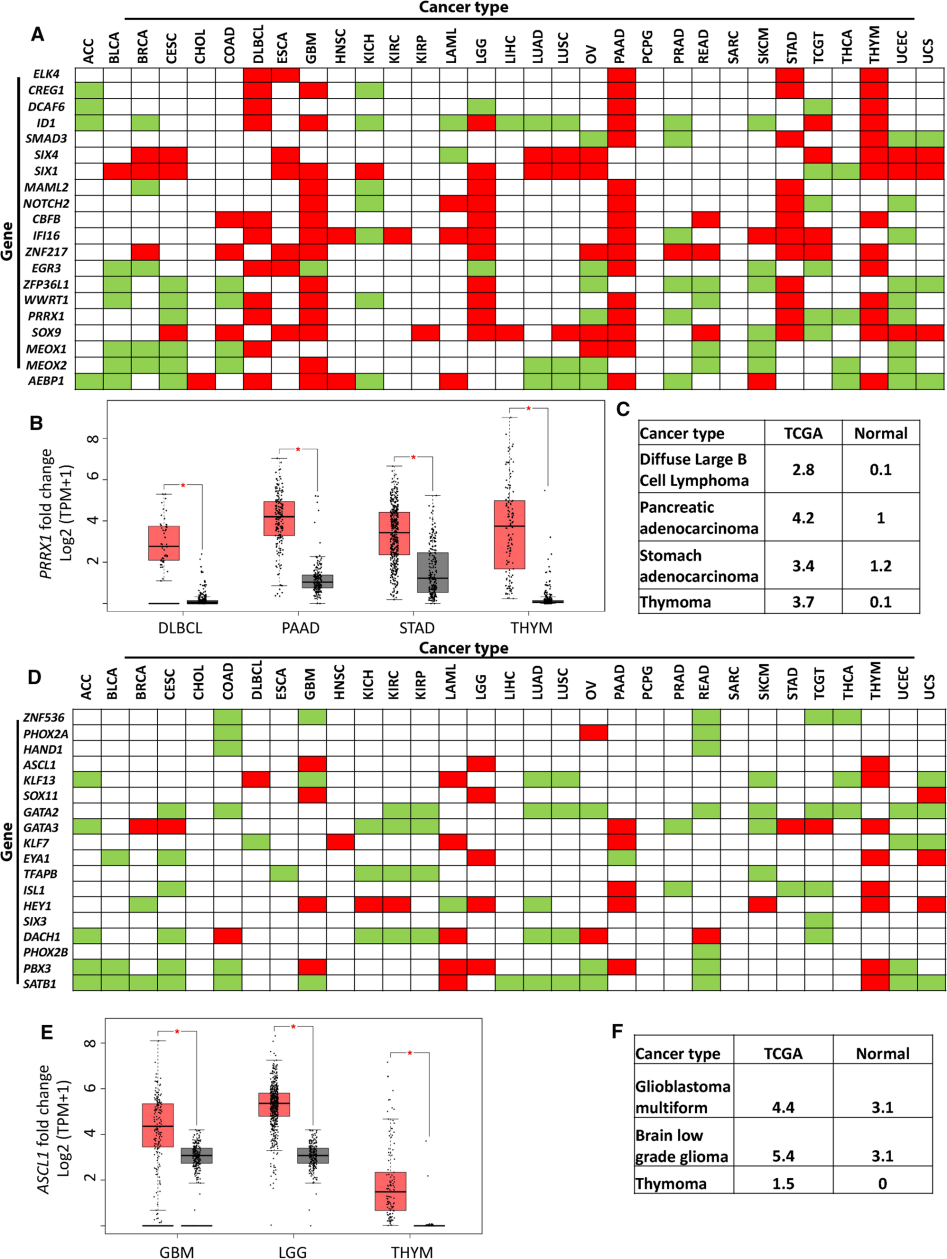
Gene expression analysis of MES TFs in 31 cancer types. **A** Expression of TFs in cancer types based on TCGA records in comparison to matched normal tissue: significant overexpression in TCGA over normal tissue is displayed in red, while overexpression in normal tissue compared to TCGA data are displayed in green. **B** *PRRX1* is significantly overexpressed in TCGA samples for diffuse large B cell Lymphoma (DLBCL), pancreatic adenocarcinoma (PAAD), thymoma (THYM) and stomach adenocarcinoma (STAD) tumours compared to normal samples expressed in log2 (TPM + 1), **C** *PRRX1* gene expression fold change in TCGA in comparison to normal samples. The parameters for this analysis were set as Log2FC cut-off of 1 and *p*-value < 0.01. One-way ANOVA was used to test for differences in expression between normal and cancer tissue. **D** Expression of TFs in cancer types based on TCGA records in comparison to matched normal tissue, significant overexpression in TCGA over normal is displayed in red, while overexpression in normal over TCGA is displayed in green. **E** *ASCL1* is significantly overexpressed in TCGA samples for glioblastoma multiforme (GBM), low grade glioma (LGG) and thymoma (THYM) tumours compared to normal samples expressed in log2 (TPM + 1), **F** *ASCL1* gene expression fold change in TCGA in comparison to normal samples. The parameters for this analysis were set as Log2FC cut-off of 1 and *p*-value < 0.01. One-way ANOVA was used to test for differences in expression between normal and cancer tissue

The expression of 20 MES and 18 ADRN CRC associated genes were analysed in over 30 cancer types using GEPIA2. Of the 20 MES TFs 11, 6, 16, 12, and 14 were significantly overexpressed in DLBCL, oesophageal squamous cell carcinoma (ESCA), pancreatic adenocarcinoma (PAAD), stomach adenocarcinoma (STAD), and thymoma (THYM) patient tumour samples, respectively, compared to matched healthy samples (Fig. 3A). For example, for DLBCL, PAAD, THYM, and STAD, *PRRX1* showed significantly higher expression levels in tumour compared to matched normal tissue (*p* < 0.01) (Fig. 3B, C.

Furthermore, of the 18 ADRN TFs 4, 5, and 8 were significantly overexpressed in glioblastoma multiforme (GBM), low-grade glioma (LGG), and THYM, respectively (Fig. 3D). For instance, *ASCl1* displayed significantly higher expression over normal tissue samples in GBM, LGG, and THYM (*p* < 0.01) (Fig. 3E, F) [52].

In addition, we linked the expression of the 38 TFs in over 30 cancer types with their mutational profile in these cancers (Additional file 1: Materials S2). For instance, we have displayed up- or downregulation of the TF genes based on the data presented in Fig. 3 and we have linked this information with various patient sample mutations for that cancer type obtained from cBioportal. Notably, GATA3 is upregulated in BRCA and we linked this to multiple putative driver mutations including *GATA3 M293K*, GATA3 S402Lfs*105, GATA3 F430Lfs*45, GATA3 L396Pfs*111, GATA3 P408Afs*99. This observation also applied to STAD in which we linked the upregulation of GATA3 with some driver mutations including GATA3 S237Afs*28 and GATA3 S237Afs*28, although these links do not necessarily convey causative effects. These results suggest a significant role for these TFs in a wider range of cancers.

### TIMER2 analysis shows an association of the 38 TFs with patient survival across cancer types

**Fig. 4:**
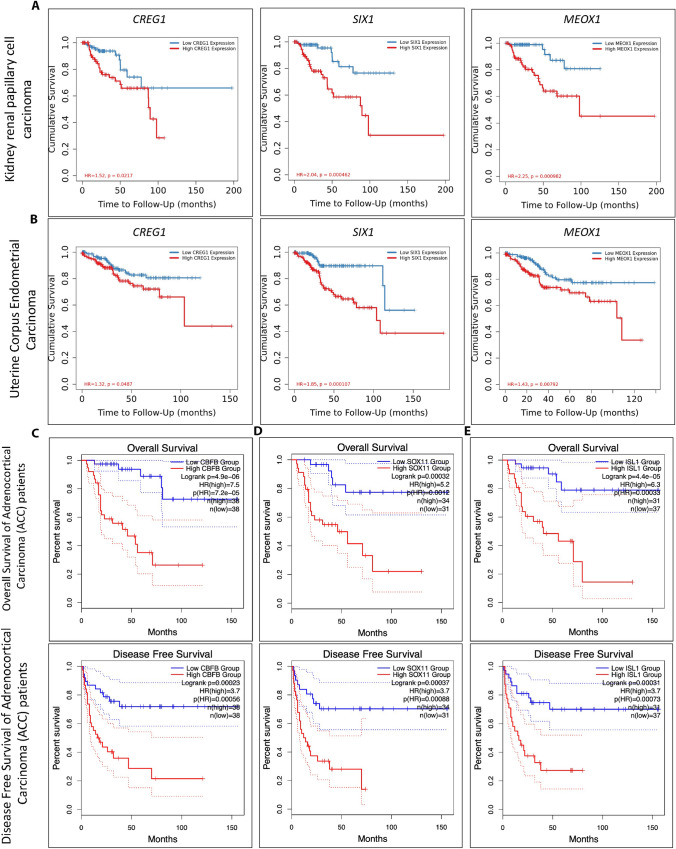
Kaplan Meier curves for cumulative survival, OS and DFS in cancer patients. The outcome of *CREG1*, *SIX1* and *MEOX1* expression in patients with KIRP and UCEC using Kaplan Meier curves. **A** For KIRP: *CREG1* (HR = 1.52, *p* = 0.0217), *SIX1* (HR = 2.04, *p* = 0.00046), and *MEOX1* (HR = 2.25, *p* = 0.00098 were obtained), **B** for UCEC: *CREG1* (HR = 1.32, *p* = 0.048), *SIX1* (HR = 1.85, *p* = 0.0001), and *MEOX1* (HR = 1.43, *p* = 0.0079) were obtained (red and blue lines representing increased and reduced expression in patients, respectively). In all cases, increased expression of these genes correlates with reduced cumulative survival of patients. **C**–**E** OS and DFS Kaplan Meier curves generated by GEPIA2 for ACC patients for *CBFB* (top: log-rank test *p* = 4.9E-06, HR = 7.5, *p*(HR) = 7.2E-5 and bottom: log-rank test *p* = 0.00023, HR = 3.7, *p*(HR) = 0.00056), *SOX11* (top: log-rank test *p* = 0.00032, HR = 5.2, *p*(HR) = 0.0012 and bottom: log-rank test *p* = 0.00037, HR = 3.7, *p*(HR) = 0.00088), and *ISL1* (top: log-rank test *p* = 4.4E-05, HR = 6.3, *p*(HR) = 0.00033 and bottom: log-rank test *p* = 0.00031, HR = 3.7, *p*(HR) = 0.00073) revealing significant decreases in OS and DFS for patients. HR = Hazard risk

Kaplan Meier survival analysis was conducted for 20 MES and 18 ADRN genes in over 30 cancer types and their subtypes using the TIMER 2 gene_outcome module. This tool uses a Cox proportional hazard (PH) to assess the clinical significance of the expression of a gene in a cancer type in the form of Kaplan Meier OS analysis (Additional file 1: Materials S3). For example, for 290 Kidney renal papillary cell carcinoma (KIRP) and 545 Uterine corpus endometrial carcinoma (UCEC) patients, the expression of 16/38 and 12/38 genes respectively (e.g., *CREG1*, *SIX1*, and *MEOX1*), was linked with a significantly higher risk and a resulting reduced OS (*p* < 0.05, Spearman’s *p* > 0) (Additional file 1: Materials S3). For instance, the cumulative survival for KIRP and UCEC patients expressing *CREG1* (HR = 1.52, *p* = 0.0217)*, SIX1* (HR = 2.04, *p* = 0.00046)*,* and *MEOX1* (HR = 2.25, *p* = 0.00098) was significantly reduced. Similarly, cumulative survival was reduced for UCEC patients, expressing *CREG1* (HR = 1.32, *p* = 0.048), *SIX1* (HR = 1.85, *p* = 0.0001), and *MEOX1* (HR=1.43, *p*=0.0079) (Fig. 4A, B. These results suggest that the 38 TFs are associated with patient survival in a wide range of cancers, highlighting overlapping mechanisms, processes, and gene networks and indicating that these could be used as biomarkers for outcome for a range of cancers.

Indeed, considering GEPIA2, survival maps for OS and DFS were generated for the 20 MES and 18 ADRN TFs over 30 cancer types (Additional file 3: Fig. S2 and Additional file 4: Fig. 3, respectively). These analyses revealed an association of the expression of these genes with OS and DFS (Additional file 3: Fig. S2 and Additional file 4: Fig. 3). For example, significantly higher risk and consequent reduced OS and DFS were detected for KIRP patients expressing *MEOX1* and *MEOX2* (Additional file 3: Fig. S2) [53]. A similar result was also found for adrenocortical adenocarcinoma (ACC) patients expressing *GATA3, GATA2,* and *SOX11* (Additional file 4: Fig. S3) [54].

Furthermore, OS and DFS for ACC patients expressing *CBFB* (HR = 7.5, log-rank test *p* = 4.9E−06 and HR = 3.7, log-rank test *p* = 0.00023) were reduced (Fig. 4C, top and bottom). Similarly, OS and DFS for ACC patients expressing *SOX11* (HR = 5.2, log-rank test *p* = 0.00032 and HR = 3.7, log-rank test *p* = 0.00037) (Fig. 4D, top and bottom) and *ISL1* (HR = 6.3, log-rank test *p* =4.4E−05 and HR = 3.7, log-rank test *p* = 0.00031) were also reduced (Fig. 4E, top and bottom). These data reveal a lower OS and DFS for ACC patients expressing *CBFB, SOX11,* and *ISl1* individually, demonstrating the significance of these genes not only in NB but also other solid cancers. Given these results, we sought to investigate the association of these TFs with patient prognosis using other databases.

### Human pathology atlas analysis of the 38 TFs shows their association with prognosis in cancers

**Fig. 5:**
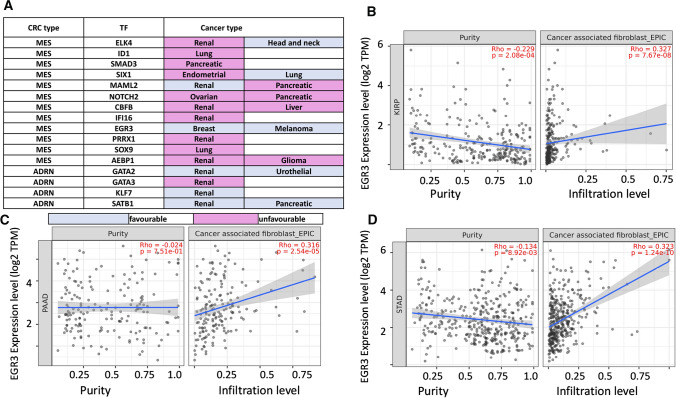
Prognostic summary and immune cell infiltration associated with MES and ADRN TFs. **A** Data obtained from the human pathology atlas reveals a significant association between the expression of MES and ADRN TFs providing either favourable or unfavourable predictive value in various cancers as indicated (pink and blue colours represent unfavourable and favourable prognosis, respectively). The classification of favourable and unfavourable prognosis in this database, is based on calculated survival probability expressed in respective Kaplan Meier curves. **B** Positive association between EGR3 expression with CAF infiltration in the TME in KIRP (*p* = 7.67E-08, Spearmann’s Rho = 0.327), **C** PAAD (*p* = 2.54E-05, Spearmann’s Rho = 0.316) and **D** STAD (*p* = 1.24E-10, Spearmann’s Rho = 0.323). For all plots, EPIC estimations for expression are displayed as log2 TPM and were adjusted for tumour purity. EPIC estimations allowed for the comparison of cell types within a sample and provided scores for cell fractions. CRC = Core regulatory circuitry, TF = Transcription Factor

We found an association of the 38 TFs with prognosis (both favourable and unfavourable) in various cancers including renal, lung, pancreatic, endometrial, ovarian, liver, breast, glial, urothelial, and melanoma (Fig. 5A). For instance, *ELK4, CBFB, IFI16, PRRX1, AEBP1,* and *GATA3* expression was associated with an unfavourable outcome in renal cancer revealing the significance of these TFs (pink and blue colours represent unfavourable and favourable prognosis, respectively) [55] (Fig. 5A).

Further to the association of expression of these TFs with patient survival, previous studies have linked their expression with various immune cells in the TME [27], leading us to address the impact of MES and ADRN TFs on the TME.

### Immune cell infiltration correlates with gene expression across cancer types

Using TIMER2 to associate gene expression with immune cell infiltration, we compared Tregs, CAFs, ECs, B cells, and gamma-delta T cells with 20 MES and 18 ADRN genes for KIRP, ACC, DLBCL, PAAD, THYM, STAD, and GBM adjusting for tumour purity. We found a strong positive association (*p* < 0.05, Spearman's Rho > 0) between the expression of multiple MES and ADRN TFs with the infiltration of CAFs, ECs, and B cells in KIRP and PAAD, while this only applied to CAFs and ECs in STAD and THYM (Additional file 1: Materials S4). Further, the expression of these TFs in ACC and DLBCL showed a negative association with Treg cells (*p* < 0.05, Spearman's Rho < 0), while the picture for gamma-delta T cells was mixed (Additional file 1: Materials S4).

A positive association of expression of EGR3 with CAFs in KIRP (*p* = 7.67E−08, Spearmann’s Rho = 0.327), PAAD (*p* = 2.54E−05, Spearmann’s Rho=0.316) and STAD (*p* = 1.24E−10, Spearmann’s Rho = 0.323) was detected (Fig. 5B, D). These results suggest that the expression of EGR3 in KIRP, PAAD, and STAD shows a strong positive correlation with TME elements, including stromal and immune cells [56]. Accordingly, the interaction of stromal cells such as CAFs with tumour cells in the TME can shape the immunosuppressive microenvironment and tumour-promoting phenotypes and potentially inform on therapeutic strategies that aim to reverse CAF-mediated immunosuppression [57]. Hence, the positive association of *EGR3* gene expression with CAFs in these cancers suggests the contribution of this gene to promoting the immunosuppressive roles of CAFs, while negative correlations obtained for Tregs in DLCBCL would suggest the reverse. Moreover, different components of the TME, including tumour-associated macrophages (TAMs), induce EMT and cancer cell migration, while EMT, chiefly triggered by hypoxia can activate stemness factors [33]. Hence, we sought to understand the influence of these TFs on the occurrence of EMT and the expression of stemness factors.

### MES and ADRN TFs are strongly associated with EMT and stemness factors across cancers

Given the link between MES and ADRN TFs and cancer cell migration and the activation of stemness factors [33, 57], we used the TIMER2 gene correlation module to establish such associations, detailed further in Additional file 1: Materials S5. For example, a positive correlation with all markers tested including CD44, CDH1, CDH2, FN1, FOXC2, NANOG, SOX2, TWIST1, and VIM was observed with the following MES and ADRN TFs: SMAD3, CBFB, ZFP36L, KLF7, DACH1, and SATB1 in liver hepatocellular carcinoma (LIHC). This positive association implies that SMAD3 may contribute to EMT and stemness programmes in these cancers, potentially contributing to their aggressiveness and metastatic potential. Previous studies reported the role of TGF-β/SMAD signalling in inducing both stemness markers and EMT in prostate cancer cells by post-transcriptional modification of CD44 [58]. These findings collectively suggest that MES and ADRN TFs also show a positive association with genes involved in EMT and cancer stemness characteristics in other solid cancers. Thus, we compared the extended network comprising TF mRNAs and miRNAs that influence the TME, EMT, stemness markers, and aggressive behaviour in cancers.

### Analysis of TF-gene networks, integration, and visualisation of network data shows that these extended networks influenced the TME and cancer progression

**Fig. 6:**
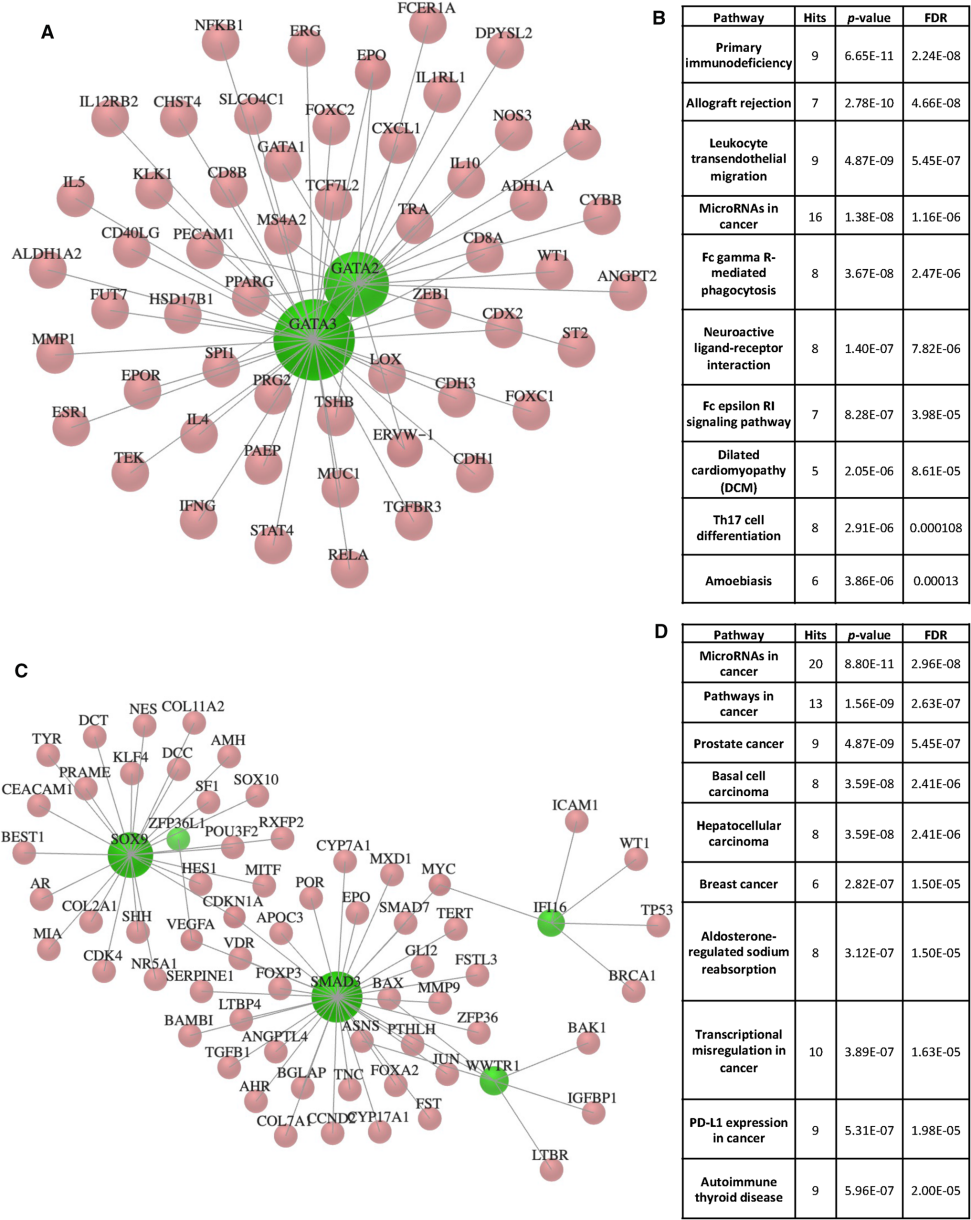
Network connectivity of MES and ADRN TFs.** A** GATA3 and GATA2 display the highest degree of connectivity with 38 and 19 connections, respectively.** B** KEGG gene enrichment analysis reveals enrichment for ‘immune’ and ‘miRNA’ in cancer terms.** C** SMAD3, SOX9, WWTR1 and IFI16 have 32, 23, 6 and 5 connections, respectively,** D** KEGG gene enrichment analysis revealed enrichment for ‘breast cancer’, ‘prostate cancer’ and ‘hepatocellular cancer’ in addition to ‘miRNAs in cancer’ terms

We sought to determine the extended networks of the 38 TFs and miRNA in impacting the TME, progression, and aggressive behaviour of cancers by using software packages including Omicsnet, Cytoscape, and dbCoRC. GATA3 and GATA2 displayed the highest degree of connectivity with 38 and 19 connections, respectively (Fig. 6A), and KEGG gene enrichment analysis revealed enrichment for immunity functions and processes, and miRNA in cancer terms (Fig. 6B). SATB1 also showed a degree of connectivity of 15 (not shown in Fig. 6B to improve the legibility of the network). In addition, MES TFs, SMAD3, SOX9, WWTR1, and IFI16 had 32, 23, 6, and 5 connections, respectively (Fig. 6C), and KEGG gene enrichment analysis revealed enrichment for ‘breast cancer’, ‘prostate cancer’, and ‘hepatocellular carcinoma’ processes in addition to ‘miRNA in cancer’ terms (Fig. 6D). This analysis suggests that the extended network of TF-gene interactomes for MES and ADRN TFs, also play roles in cancer pathways, gene regulation, and immune-related processes.

Consistent with the miRNA links identified, we used Cytoscape to search for miRNA- mRNA networks of *GATA3* and *SOX9* in DLBCL and LIHC, respectively, as two examples of such networks (Additional file 5: Fig. S4). *GATA3* associated with TCGA data for DLBCL (GO enrichment q-value = 6.55). Also, a negative correlation of *GATA3* with *hsa-mir-431* (*p*-value = 4.9−E07, correlation = − 0.65) and *hsa-mir-433* (*p* = 3.33E−5, correlation = − 0.566) was observed (Additional file 5: Fig. S4B). Closer inspection of the network also revealed the presence of *ASCL1*, another member of the MES CRC TFs, which also displayed negative regulation with *hsa-mir-431* (*p* = 3.58E−6, correlation = − 0.61), *hsa-mir-432* (*p* = 1.7E-4, correlation = − 0.52) and *hsa-mir-433* (*p* = 1.48E–5, correlation=− 0.58) (Additional file 5: Fig. S4C). *hsa-mir-433,* a tumour suppressor in breast cancer, inhibits the growth of cancer cells by affecting cell migration [59]. Similarly, *SOX9* was associated with TCGA data for LIHC (miRNA vs RNA) (GO enrichment q-value = 6.75) (Additional file 5: Fig. S4D). SOX9 positively correlated with *hsa-mir-429* (= 4.03E−32, correlation = 0.56), *hsa-mir-200a* (*p* = 1.54E−32, correlation = 0.56) and *hsa-mir-200b* (*p* = 3.8E−30, correlation = 0.54) (Additional file 5: Fig. S4E). The role of *hsa-mir-429* as a biomarker of cancer initiation and progression for breast cancer has been previously validated, and, similarly, the *mir-200* family members have been reported as potential prognostic biomarkers in multiple cancers [60, 61]. Collectively these results suggest the intricate connectivity of genes such as GATA3 and SOX9 that play essential roles in NB differentiation and neural crest development, respectively, with networks of miRNAs. These miRNAs have prognostic value in various cancers and should be investigated further in NB [13]. In Table 1, we have provided examples of the predicted prognostic potential of the 38 TFs with NB risk groups and patient survival in NB and other cancers.

**Table 1:** Examples of associations with NB risk group or patient survival data for NB and other cancers

NB Subtype	TF	Examples of associations with NB risk group or patient survival data in NB and other cancers determined in this study
MES	ELK4	Associated with an unfavourable outcome in renal cancer patients, associated with a favourable outcome in head and neck cancer patients
MES	CREG1	Upregulated in KIRP and UCEC patients with reduced survival
MES	DCAF6	Downregulated in high-risk NB
MES	ID1	Upregulated in high-risk NB, associated with an unfavourable outcome in lung cancer patients
MES	SMAD3	Upregulated in high-risk NB, associated with an unfavourable outcome in pancreatic cancer patients
MES	SIX4	Expression in cholangiocarcinoma is associated with a higher Spearman risk and reduced survival
MES	SIX1	Upregulated in KIRP and UCEC patients with reduced survival, associated with an unfavourable outcome in endometrial cancer patients, associated with a favourable outcome in lung cancer patients
MES	MAML2	Associated with a favourable outcome in renal cancer patients, associated with an unfavourable outcome in pancreatic cancer patients
MES	NOTCH2	Associated with an unfavourable outcome in ovarian and pancreatic cancer patients
MES	CBFB	Downregulated in high-risk NB, upregulated in ACC patients with reduced survival, associated with an unfavourable outcome in renal and liver cancer patients
MES	IFI16	Associated with an unfavourable outcome in renal cancer patients
MES	ZNF217	Upregulated in high-risk NB
MES	EGR3	Upregulated in NB patients with reduced survival, upregulated in high-risk NB, associated with a favourable outcome in breast cancer and melanoma patients
MES	ZFP36L1	Expression in KIRP patients is associated with a higher Spearman risk and reduced survival
MES	WWTR1	Expression in LGG patients is associated with a higher Spearman risk and reduced survival
MES	PRRX1	Associated with an unfavourable outcome in renal cancer patients
MES	SOX9	Associated with an unfavourable outcome in lung cancer patients
MES	MEOX1	Downregulated in high-risk NB, upregulated in KIRP and UCEC patients with reduced survival
MES	MEOX2	Expression in KIRP patients is associated with a higher Spearman risk and reduced survival
MES	AEBP1	Associated with an unfavourable outcome in renal cancer and glioma patients
ADRN	ZNF536	Expression in KIRP patients is associated with a higher Spearman risk and reduced survival
ADRN	PHOX2A	Expression in STAD and UCEC patients associated with higher Spearman risk and reduced survival
ADRN	HAND1	Expression in LIHC patients is associated with a higher Spearman risk and reduced survival
ADRN	ASCL1	Upregulated in NB patients with reduced survival
ADRN	KLF13	Downregulated in high-risk NB
ADRN	SOX11	Upregulated in high-risk NB, upregulated in ACC patients with reduced survival
ADRN	GATA2	Downregulated in high-risk NB, associated with a favourable outcome in renal and urothelial cancer patients
ADRN	GATA3	Upregulated in high-risk NB (not significant), associated with an unfavourable outcome in renal cancer patients
ADRN	KLF7	Upregulated in NB patients with increased survival, downregulated in high-risk NB, associated with a favourable outcome in renal cancer patients
ADRN	EYA1	Expression in ACC patients is associated with a higher Spearman risk and reduced survival
ADRN	TFAP2B	Downregulated in high-risk NB
ADRN	ISL1	Upregulated in ACC patients with reduced survival
ADRN	HEY1	Expression in ACC patients is associated with a higher Spearman risk and reduced survival
ADRN	SIX3	Upregulated in high-risk NB
ADRN	DACH1	Expression in ACC patients is associated with a higher Spearman risk and reduced survival
ADRN	PHOX2B	Expression in GBM patients is associated with a higher Spearman risk and reduced survival
ADRN	PBX3	Upregulated in NB patients with reduced survival, downregulated in high-risk NB
ADRN	SATB1	Downregulated in high-risk NB, associated with a favourable outcome in renal and pancreatic cancer patients

Finally, we used the dbCoRC database to integrate mRNA expression of core TFs with their reconstructed circuitry in cancers. For instance, SMAD3 was indicated in the CRC of representative cell lines of DLBCL, colorectal cancer, pancreatic, hepatocellular carcinoma, breast, and gastric cancers (Table 2). These results suggested similarities in transcriptional regulatory mechanisms governed by these TFs in various cancer types [44], hence pointing towards the versatility and commonalities of these networks in cancers. These core TFs may display similar or contrasting diagnostic and prognostic values, a result warranting further investigation and validation.

**Table 2:** The integration of network connectivity of MES and ADRN TFs in CRCs of other cancers

CRC TF (MES or ADRN)	Utility of TF in cancers (an example of a corresponding cell line) determined in this study
SMAD3	DLBCL (SU-DHL-6), colorectal cancer (HCT-116), pancreatic cancer (MiaPaca2), hepatocellular carcinoma (HepG2), breast cancer (MDA231), gastric cancer (MKN7)
ZNF217	Breast cancer (MDA231), colorectal cancer (HCT-15), gastric cancer (T980436)
KLF13	Gastric cancer (T2001206), small cell lung cancer (NCI-H69)
GATA2	Prostate cancer (LNCaP)
GATA3	Breast cancer (MCF7)
KLF7	Gastric cancer (T2000085), small cell lung cancer (NCI-H69)
PHOX2B	Small cell lung cancer (NCI-H82)

## Discussion

Core regulatory circuitries can regulate lineage or cell-specific gene expression and thereby confer a specific identity. These CRCs comprise super enhancers that are marked with high deposition of permissive H3K27 acetyl histone marks that drive a set of highly regulated TFs, which in turn self-regulate and regulate the expression of other TFs within the CRC [8–11].

Two specific cell identities have been identified in NB tumours; ADRN and MES, the latter bearing higher therapy resistance and being enriched in relapsed samples [5–7]. Despite conferring specific yet interconvertible cell identities in a rare paediatric cancer by the 38 MES and ADRN TFs, we were intrigued to study these TFs in both NB and the wider context of cancers to assess their potential as indicators of disease prognosis, progression, and relapse. Accordingly, MES-specific minimal residual disease (MRD) markers in NB were identified as *PRRX1, EMO3,* and *POSTN* in previous studies [62]. The detection of the mRNA of these genes in peripheral blood was effectively used for assessing MRD and correlated with low OS and DFS in NB patients, collectively suggesting the potential use of PRRX1 as a biomarker for prognosis, treatment, and remission in NB [62]. On a similar note, in several reports, GATA2 was shown to be associated with low-risk disease and better NB patient prognosis [63, 64], while EYA1 was expressed in earlier stages of NB [24]. Given this background, we aimed to dissect such links more comprehensively in NB and other cancers, specifically from the perspective of MES- and ADRN-specific CRC TFs. To that end, the extended list of MES and ADRN genes were initially subjected to cBioportal for NB samples retrieving identified genetic alterations with unknown or predicted oncogenic functions, including amplification, missense, in-frame, and truncating mutations, in addition to fusions and deletions in 853 genes, with the vast majority bearing very low mutation rates per sample (< 0.5%). The identification and study of potential drivers in cancers will not only lead to the greater dissection of genomic alterations but may also inform future diagnostic and therapeutic studies. Gene Ontology enrichment studies revealed the enrichment of JAK/ STAT signalling for the MES gene list perhaps suggesting a requirement for the signalling input from this pathway in cell proliferation, tumourigenesis, and migration [65–70]. Furthermore, we elected to focus on the list of 20 MES and 18 ADRN CRC TFs, initially testing the expression of MES and ADRN genes with NB risk levels. This study revealed that ADRN-associated *SATB1*, *GATA2*, *TFAP2B, KLF13*, *KLF7,* and *PBX3* were downregulated in high-risk NB cases, while *SIX3* and *GATA3* were upregulated in this group. Similarly, MES-associated *MEOX1, CBFB,* and *DCAF6* genes were downregulated in high-risk NB cases, while *SMAD3, ID1*, *SOX11, ZNF217,* and *EGR3* were upregulated. Evidence supports the potential of *GATA2* as an early cancer detection marker and a good prognostic factor in NB, with high *GATA2-*expressing patient groups displaying greater OS compared to those with low levels of *GATA2* [22, 63, 64]. Consistently, we showed that GATA2 was downregulated in high-risk NB. Consistently, ID1, SOX11, TFAP2B, KLF7, and GATA3 were linked to neurogenesis or NB differentiation [13, 18, 19, 23, 71, 72], but to our knowledge, these studies did not discuss their association with survival and risk groups in NB, hence our findings are novel.

Evidence suggested the importance of lncRNAs in NB risk group stratification, leading us to compare the network formed by the 38 TFs and lncRNAs expression [46]. We identified that *DBH-AS1* expression was strongly associated with *TFAP2B* and *GATA2*. Furthermore, its expression was lower in high-risk NB and was associated with better prognosis. In keeping with these data, the downregulation of *DBH-AS1* in osteosarcoma was shown to be an indicator of good prognosis in these patients [73], amongst other reports in the literature highlighting the significance of this lncRNA in various cancers [48, 74].

Given the connection between the 38 TFs and the reported lncRNAs in other cancers [48–51], we elected to expand our investigation to other cancers. We used several bioinformatic tools, including GEPIA2, TIMER2, Omicsnet, Cytoscape, and dbCoRC to gain a deeper understanding of the role of these TFs in other cancers including their overexpression, prognostic value, association with cells of the TME, miRNA-TF network connectivity, and utility in other CRCs. Both MES and ADRN TFs displayed patterns of overexpression in TCGA tumour samples compared to matched normal samples. For instance, MES-specific PRRX1 (sufficient to convert ADRN to MES subtypes) [5, 6] was overexpressed in tumour samples compared to matched normal tissue of multiple cancers including low-grade glioma, GBM, DLBCL, STAD, PAAD, and THYM. Consistent with this finding, the role of PRRX1 in promoting stemness and angiogenesis in glioma has been reported [75]. In our study, ADRN-specific GATA3 was overexpressed in breast invasive carcinoma (BRCA), cervical squamous cell carcinoma and endocervical adenocarcinoma (CESC), testicular germ cell tumours (TGCT), STAD, PAAD, and THYM, data that we matched with the mutational profiles of these genes. Consistent with these findings, the predictive value of SMAD4/GATA3 for OS and relapse-free survival (RFS) for breast invasive ductal carcinoma has been reported in previous studies with SMAD4-/GATA3+ patients displaying better OS and RFS [76].

The association of these TFs with lower survival was determined using the TIMER2 tool across various cancer types. For more than half of the MES and ADRN TFs (e.g., SIX1, MEOX1, and ZNF536), decreased cumulative survival was observed for two cancer types in particular; KIRP and UCEC, but to a lower extent in other cancers, indicating the influence of these TFs on patient survival in these cancers. Consistently, the high expression of SIX1 in endometrial cancer could be a predictor of unfavourable prognosis in these patients [77]. Similarly, higher Spearman’s risk and reduced survival were observed for LGG, STAD, mesothelioma (MESO), pheochromocytoma and paraganglioma (PCPG), and thyroid carcinoma (THCA) patients expressing SIX1, collectively suggesting the prognostic value of SIX1 in these cancers. On a similar note, a significant association between the expression of ELK4, CBFB, IFI16, PRRX1, AEBP1, and GATA3 with an unfavourable outcome in renal cancer has been identified in previous reports [78, 79]. In light of these findings, the most significant result obtained in this study is the potential prognostic values of the 38 TFs across the cancer types reported in Table 1.

We next investigated the association between MES and ADRN TFs with the infiltration of cells associated with cells in the TME including Treg, CAFs, ECs, B cells, and gamma-delta T cells. We found a positive correlation of MES and ADRN TFs with the infiltration of CAFs and ECs in KIRP, PAAD, STAD, and THYM. Recent studies have suggested that CAFs produce growth factors and cytokines that may facilitate angiogenesis, promote tumour growth and modulate cancer stem cell characteristics [80]. In keeping, the role of CAFs in promoting angiogenesis in gastric cancer has previously been reported [81]. We also found strong correlations between MES and ADRN TFs with markers of cancer cell stemness and cancer cell motility including CDH1, CDH2, NANOG, SOX2, TWIST1, VIMENTIN, and Fibronectin across cancers. For instance, we found positive correlations between SOX9 and SATB1 with these markers in colon adenocarcinoma (COAD) and lung adenocarcinoma (LUAD), respectively. Accordingly, SATB1 promotes metastasis and cell growth in colorectal cancer [82]. In another study, TGF-β secreted by TAMs was shown to promote metastasis in non-small cell lung cancer (NSCLC) through promoting TGF-β/SOX9 axis expression [83]. These observations suggest that MES TFs may be associated with genes that mediate tumour cell motility and metastasis.

Network connectivity followed by KEGG term enrichment of MES TFs revealed that the extended network of genes linked to TFs such as SMAD3, IFI16, and SOX9 was also involved in various cancer regulatory pathways, cancer types, and transcriptional misregulation, while the extended network of GATA2 and GATA3 connected genes has roles in various immune pathways and miRNAs in cancers. Recent studies have shown that SMAD3 can have a dual role in repressing and promoting cancer by inhibiting cell proliferation and regulating transcriptional output favouring metastasis, respectively [84]. On the other hand, a study identified GATA3 upregulation and its association with favourable outcomes in breast cancer [85]. Moreover, we reveal that both GATA3 and SOX9 are negatively and positively associated with *hsa-mir-433* in DLBCL and *hsa-mir-429* in LIHC, respectively, suggesting crucial CRC TFs may overlap in function in various cancer types, govern similar regulatory networks, and display similar roles as disease biomarkers and prognostic predictors. Finally, we show that SMAD3, ZNF217, KLF13, GATA2, GATA3, KLF7, and PHOX2B are components of CRCs in other cancers, including DLBCL, pancreatic, gastric, breast, and small cell lung cancer. These data suggest similarities between CRCs and transcriptional regulatory circuitries across the malignant spectrum and that these key TFs, embedded in similar networks in various cancers, may display valuable diagnostic and prognostic factors [86].

## Conclusions

In conclusion, this study provides an overview of the implications of MES and ADRN TFs in NB risk groups and patient survival, and reveals their extended networks formed with miRNAs and lncRNAs. We also provide a pan-cancer view of the network connectivity and utility of these TFs in gene networks in other cancers and their correlation with DFS and OS, association with immune cell infiltration and stemness, and EMT markers. Our meta-analysis reveals the pan-cancer implication of the NB MES and ADRN TFs and their roles as putative prognostic predictors in various cancers. A better understanding of these genes in both NB and other cancers may pave the way to discovering specific biomarkers of disease progression, treatment response, and remission in these cancers, impacting patient survival and quality of life.

## Supplementary Information

**Additional file 1: Materials S1.** Additional file, sheet 1, 2, 3, 4 and 5 (S1, S2, S3, S3, S4 and S5, respectively)

**Additional file 2: Figure S1.** ADRN and MES TF expression correlates with NB patient OS and with lncRNAs. A) Of the TFs correlating with NB risk, *KLF7* is associated with NB patient survival based on RNA sequencing data from TARGET (red and blue lines indicating patients expressing or not expressing the indicated gene, respectively), while *HAND1* and *TFAP2B* show trends for up- and downregulation in patients with reduced survival, B) 5 TFs correlate with NB risk, based on Agilent microarray data (red and blue lines indicating patients expressing or not expressing the indicated gene, respectively), *TFAP2B* shows trends for upregulation in patients with increased survival, (C) TF-lncRNA expression correlations, for instance *PHOX2B* positively correlates with *MORC2-AS1*, D) LncRNAs associate with NB risk groups, for example, *LIFR-AS1* is downregulated in high-risk NB.

**Additional file 3: Figure S2.** Survival significance maps for OS and DFS. Survival maps for 20 MES TFs pan-cancer. The higher expression of the TFs with OS and DFS is displayed in hazard risk (HR) logarithmic values in red and blue conveying high and low survival, respectively. OS is defined as the duration since diagnosis for which the patients are still alive, while DFS is defined as the duration since treatment for which no signs of cancer are present. Cancer types are indicated on the upper x-axis and gene names on the right y-axis.

**Additional file 4: Figure S3.** Survival significance maps for OS and DFS. Survival maps for 18 ADRN TFs pan-cancer. The higher expression of the TFs with OS and DFS is displayed in hazard risk (HR) logarithmic values in red and blue conveying high and low survival, respectively. OS is defined as the duration since diagnosis for which the patients are still alive, while DFS is defined as the duration since treatment for which no signs of cancer are present. Cancer types are indicated on the upper x-axis and gene names on the right y-axis.

**Additional file 5: Figure S4.** Cytoscape analysis of GATA3, ASCL1 and SOX9. A) Cytoscape analysis of GATA3 in TCGA-DLBCL data. Exporting these network data to NDEx facilitated the visualisation of the network and interrogation of 1- step neighbourhood interactions for GATA3 with other genes and miRNAs with a GO enrichment q value of 6.55. This option returned the connected nodes for the query and the edges between the nodes of the subnetwork. In this network, positive and negative correlation are depicted by red and blue edges, respectively. B) The negative correlation of GATA3 with *hsa-mir-431* (*p* = 4.9-E07, correlation: − 0.65) and *hsa-mir-433* (*p* = 3.33E−5, correlation: − 0.566). C) ASCL1, also displays a negative regulation with *hsa-mir-431* (*p* = 3.58E-6, correlation: − 0.61), *hsa-mir-432* (*p* = 1.7E−4, correlation: − 0.52) and *hsa-mir-433* (*p* = 1.48E–5, correlation: − 0.58), D) SOX9 in TCGA-LIHC data with a GO enrichment q-value of 6.75. E) SOX9 positively correlates with *hsa-mir-429* (*p* = 4.03E−32, correlation: 0.56), *has-mir-200a* (*p* = 1.54E−32, correlation: 0.56) and *has-mir-200b* (*p* = 3.8E−30, correlation: 0.54).

## Data Availability

All data generated or analysed in this study are included in this published article and its additional files.

## Abbreviations

ACC: Adrenocortical carcinoma
BLCA: Bladder Urothelial Carcinoma
BRCA: Breast invasive carcinoma
CESC: Cervical squamous cell carcinoma and endocervical adenocarcinoma
CHOL: Cholangiocarcinoma
COAD: Colon adenocarcinoma
CRC: Core regulatory circuitry
DLBC: Lymphoid Neoplasm Diffuse Large B-cell Lymphoma
DFS: Disease-free survival
ECs: Endothelial cells
EFS: Event-free survival
EMT: Epithelial to mesenchymal transition
ESCA: Oesophageal carcinoma
GBM: Glioblastoma multiforme
HNSC: Head and Neck squamous cell carcinoma
HR: Hazard risk
INRG: International Neuroblastoma Risk Group
KICH: Kidney Chromophobe
KIRC: Kidney renal clear cell carcinoma
KIRP: Kidney renal papillary cell carcinoma
LAML: Acute Myeloid Leukaemia
LGG: Brain Lower Grade Glioma
LIHC: Liver hepatocellular carcinoma
LncRNAs: Long non-coding RNAs
LUAD: Lung adenocarcinoma
LUSC: Lung squamous cell carcinoma
MESO: Mesothelioma
miRNAs: MicroRNAs
MRD: Minimal residual disease
OS: Overall survival
OV: Ovarian serous cystadenocarcinoma
PAAD: Pancreatic adenocarcinoma
PCPG: Pheochromocytoma and Paraganglioma
PRAD: Prostate adenocarcinoma
READ: Rectum adenocarcinoma
RFS: Relapse-free survival
RPKM: Reads per kilobase of transcripts per million reads
SARC: Sarcoma
SKCM: Skin Cutaneous Melanoma
STAD: Stomach adenocarcinoma
TAMs: Tumour associated macrophages
TFs: Transcription factors
TGCT: Testicular Germ Cell Tumour
THCA: Thyroid carcinoma
TME: Tumour microenvironment
TPM: Transcript count per million
Treg: T regulatory cells
UCEC: Uterine Corpus Endometrial Carcinoma
UCS: Uterine Carcinosarcoma
UVM: Uveal Melanoma
