# Supplementary figures and images for: Deep analysis of neuroblastoma core regulatory circuitries using online databases and integrated bioinformatics shows their pan-cancer roles as prognostic predictors
Source: Discov Oncol. 2021 Nov 29;12:56. doi: 10.1007/s12672-021-00452-3 (PMC8777518; doi:10.1007/s12672-021-00452-3)

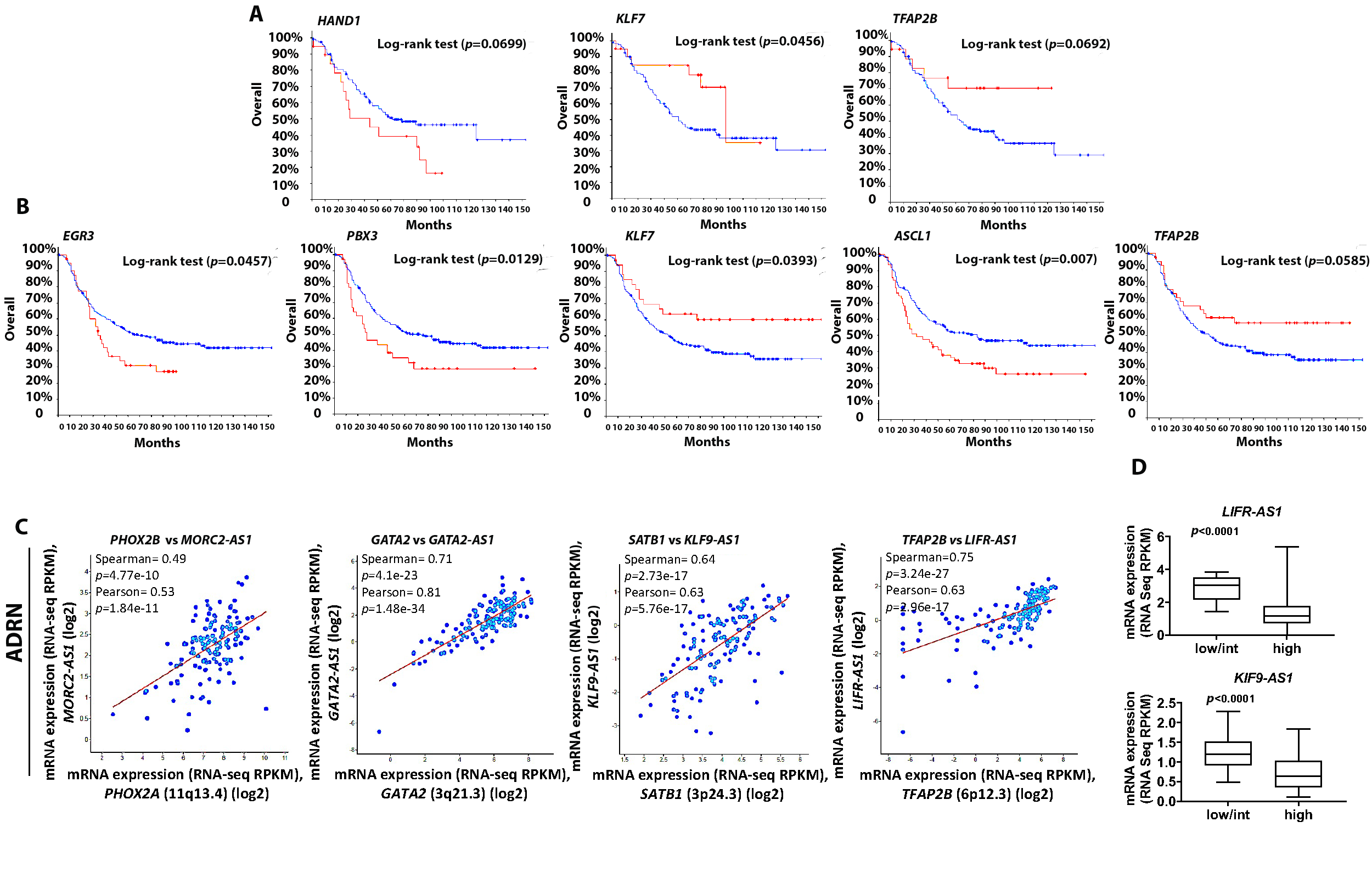

Supplement: Supplementary file 2 — Additional file 2: Figure S1. ADRN and MES TF expression correlates with NB patient OS and with lncRNAs. A) Of the TFs correlating with NB risk, KLF7 is associated with NB patient survival based on RNA sequencing data from TARGET (red and blue lines indicating patients expressing or not expressing the indicated gene, respectively), while HAND1 and TFAP2B show trends for up- and downregulation in patients with reduced survival, B) 5 TFs correlate with NB risk, based on Agilent microarray data (red and blue lines indicating patients expressing or not expressing the indicated gene, respectively), TFAP2B shows trends for upregulation in patients with increased survival, (C) TF-lncRNA expression correlations, for instance PHOX2B positively correlates with MORC2-AS1, D) LncRNAs associate with NB risk groups, for example, LIFR-AS1 is downregulated in high-risk NB. [file 12672_2021_452_MOESM2_ESM.tif]

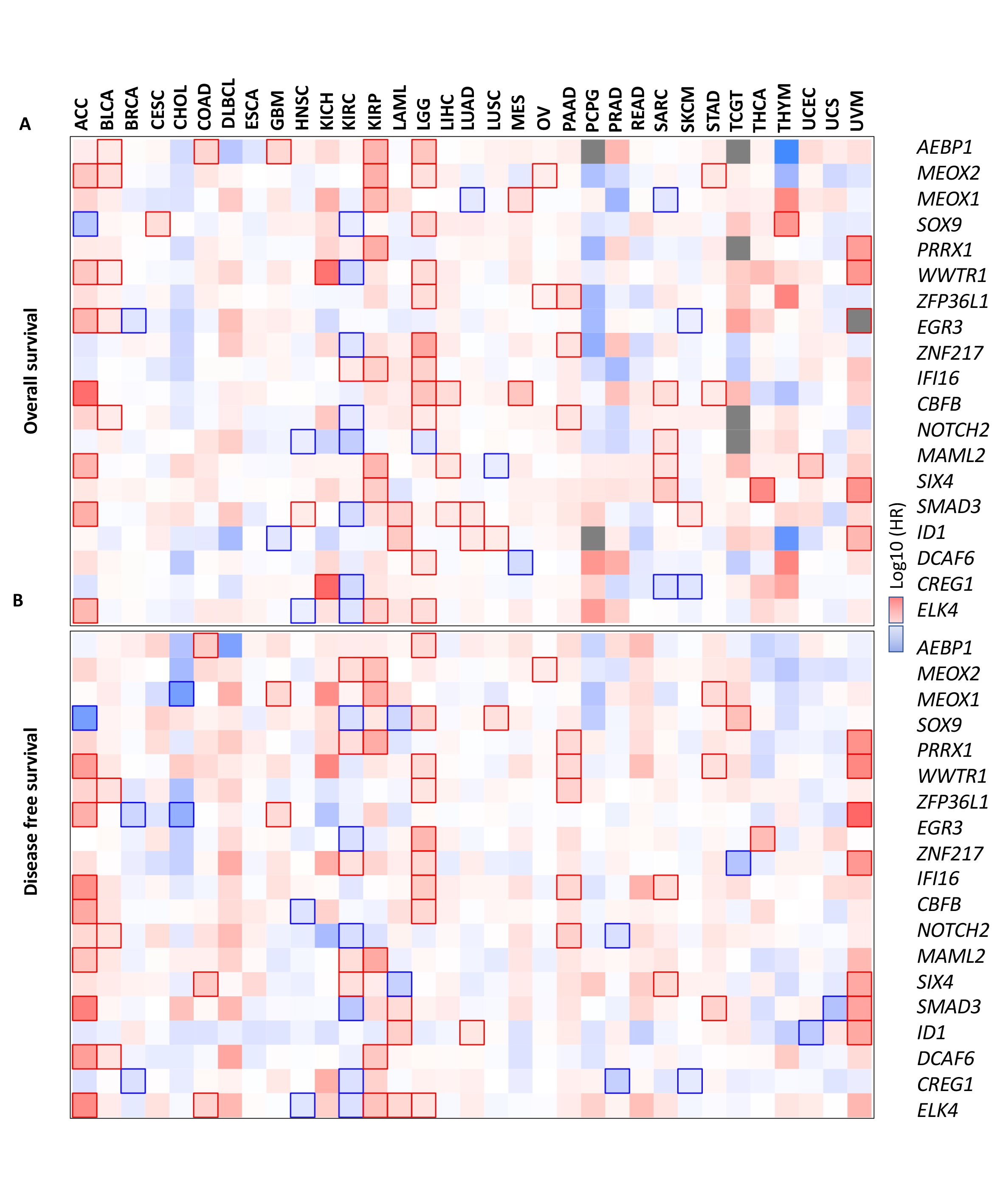

Supplement: Supplementary file 3 — Additional file 3: Figure S2. Survival significance maps for OS and DFS. Survival maps for 20 MES TFs pan-cancer. The higher expression of the TFs with OS and DFS is displayed in hazard risk (HR) logarithmic values in red and blue conveying high and low survival, respectively. OS is defined as the duration since diagnosis for which the patients are still alive, while DFS is defined as the duration since treatment for which no signs of cancer are present. Cancer types are indicated on the upper x-axis and gene names on the right y-axis. [file 12672_2021_452_MOESM3_ESM.tif]

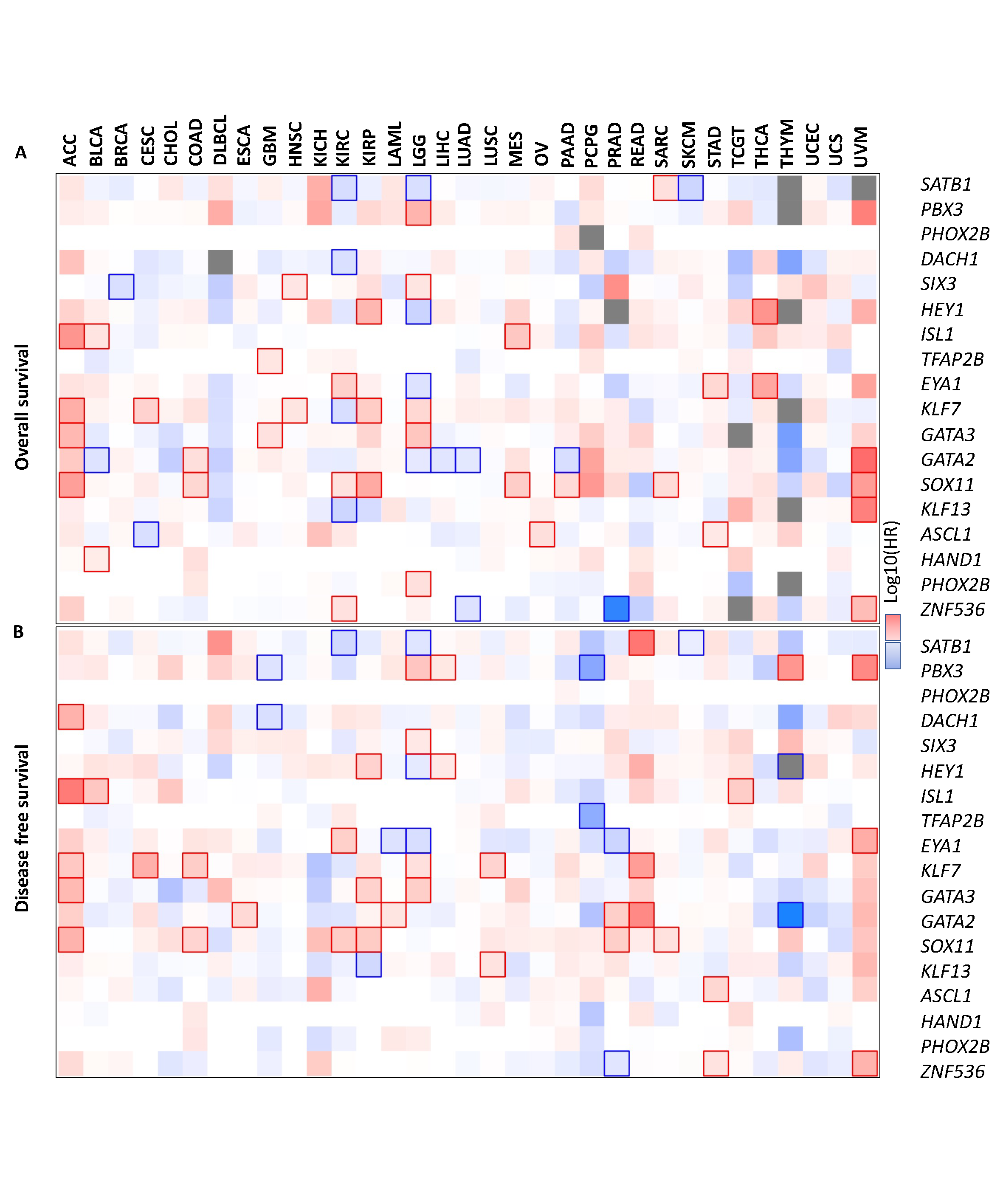

Supplement: Supplementary file 4 — Additional file 4: Figure S3. Survival significance maps for OS and DFS. Survival maps for 18 ADRN TFs pan-cancer. The higher expression of the TFs with OS and DFS is displayed in hazard risk (HR) logarithmic values in red and blue conveying high and low survival, respectively. OS is defined as the duration since diagnosis for which the patients are still alive, while DFS is defined as the duration since treatment for which no signs of cancer are present. Cancer types are indicated on the upper x-axis and gene names on the right y-axis. [file 12672_2021_452_MOESM4_ESM.tif]

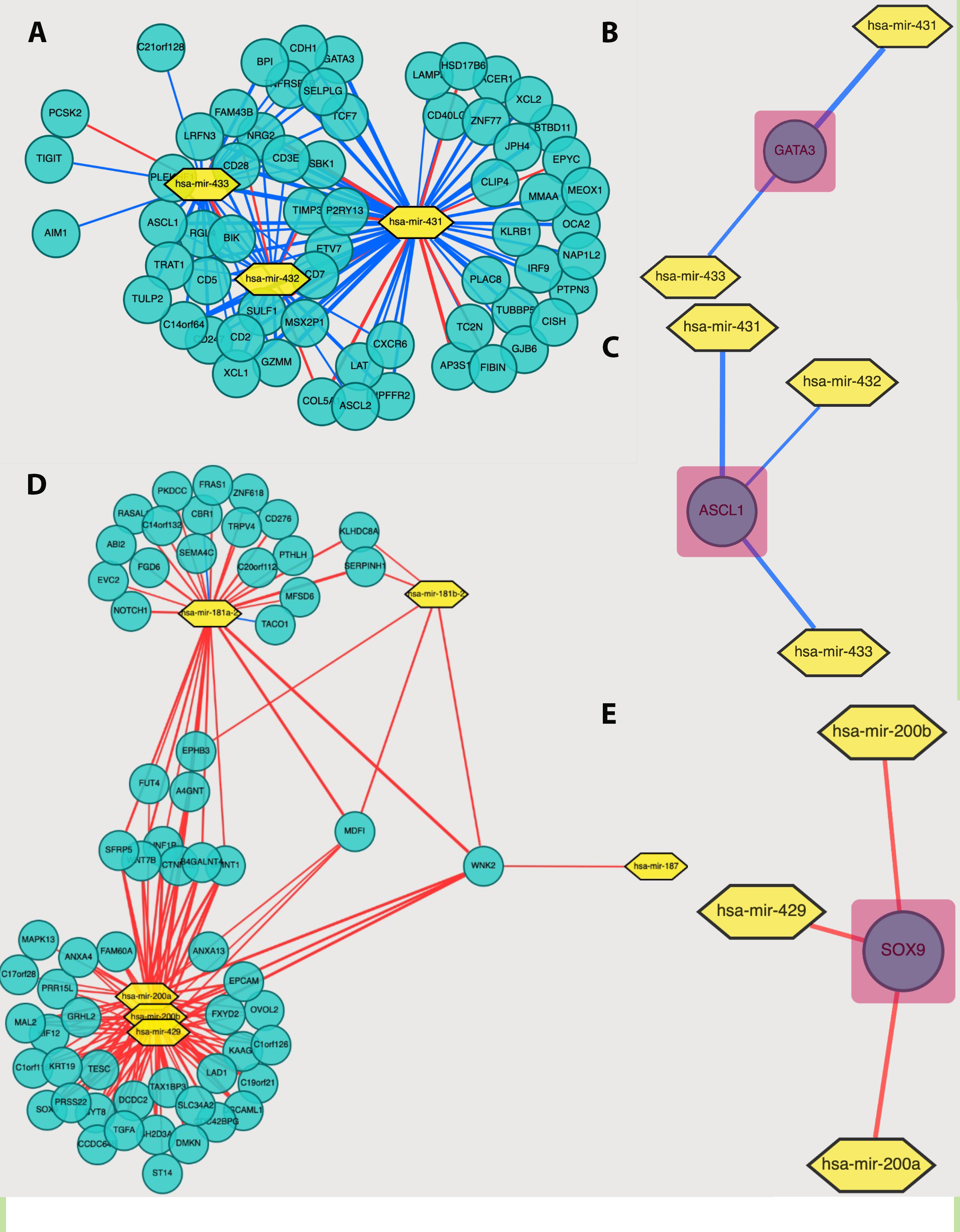

Supplement: Supplementary file 5 — Additional file 5: Figure S4. Cytoscape analysis of GATA3, ASCL1 and SOX9. A) Cytoscape analysis of GATA3 in TCGA-DLBCL data. Exporting these network data to NDEx facilitated the visualisation of the network and interrogation of 1- step neighbourhood interactions for GATA3 with other genes and miRNAs with a GO enrichment q value of 6.55. This option returned the connected nodes for the query and the edges between the nodes of the subnetwork. In this network, positive and negative correlation are depicted by red and blue edges, respectively. B) The negative correlation of GATA3 with hsa-mir-431 (p = 4.9-E07, correlation: − 0.65) and hsa-mir-433 (p = 3.33E−5, correlation: − 0.566). C) ASCL1, also displays a negative regulation with hsa-mir-431 (p = 3.58E-6, correlation: − 0.61), hsa-mir-432 (p = 1.7E−4, correlation: − 0.52) and hsa-mir-433 (p = 1.48E–5, correlation: − 0.58), D) SOX9 in TCGA-LIHC data with a GO enrichment q-value of 6.75. E) SOX9 positively correlates with hsa-mir-429 (p = 4.03E−32, correlation: 0.56), has-mir-200a (p = 1.54E−32, correlation: 0.56) and has-mir-200b (p = 3.8E−30, correlation: 0.54). [file 12672_2021_452_MOESM5_ESM.tif]
